# Epidemiology of malaria and chloroquine resistance in Mizoram, northeastern India, a malaria-endemic region bordering Myanmar

**DOI:** 10.1186/s12936-020-03170-3

**Published:** 2020-02-27

**Authors:** Rita Zomuanpuii, Christopher L. Hmar, Khawlhring Lallawmzuala, Lal Hlimpuia, Praveen Balabaskaran Nina, Nachimuthu Senthil Kumar

**Affiliations:** 1 Department of Biotechnology, Mizoram University, Aizawl, Mizoram India; 2 Department of Orthopaedics, District Hospital, Government of Mizoram, Serchhip, Aizawl, Mizoram India; 3 Department of Medicine, District Hospital, Government of Mizoram, Serchhip, Aizawl, Mizoram India; 4 State Vector Disease Control Programme, Department of Health and Family Welfare, Government of Mizoram, Aizawl, Mizoram India; 5 Department of Epidemiology and Public Health, Central University of Tamil Nadu, Tiruvarur, Tamil Nadu India

**Keywords:** Mizoram, *Plasmodium falciparum*, Chloroquine resistance, *pfcrt*, *pfmdr1*

## Abstract

**Background:**

Mizoram, a northeastern state in India, shares international borders with Myanmar and Bangladesh and is considered to be one of the key routes through which drug-resistant parasites of Southeast Asia enter mainland India. Despite its strategic location and importance, malaria epidemiology and molecular status of chloroquine resistance had not been well documented, and since chloroquine (CQ), as the first-line treatment in *Plasmodium falciparum* infection was discontinued since 2008, it was expected that CQ-sensitive haplotype would be more abundant.

**Methods:**

Malaria epidemiology data for the period 2010 to 2018 was collected from the office of State Vector Disease Control Programme. *Plasmodium falciparum*-positive blood samples were collected from government district hospitals, community health centres, primary health centres, sub-centres, and diagnostic centres from six malaria-prone districts. The samples were processed and analysed using genes–*P. falciparum* chloroquine-resistant transporter (*pfcrt*) and *P. falciparum* multidrug resistance 1 (*pfmdr1*) via sequencing of PCR amplicon from 2015 to 2017.

**Results:**

Malaria occurred throughout the year and *P. falciparum* accounted for > 89% of total malaria cases. During 2010–2018, the highest number of malaria incidence was recorded in Lawngtlai (36% of total malaria cases; average API_2010–2018_ of 34.8) while Champhai remained consistently low (0.4%; average API_2010–2018_ of 0.04). Males of ≥ 15 years old contributed maximum (35.7%) among gender and age malarial distribution recorded during 2014–2018. Death due to malaria gradually decreased over the years. A higher abundance of mutated *pfcrt* (58.5% of the total sample analysed) and a lower prevalence of mutated *pfmdr1* (48.7%) were observed. All mutations identified for *pfcrt* belong to the Southeast Asian CVIET haplotype. Only a single point mutation was observed at 86 (N → Y) position in *pfmdr1* (48.7%). The key N86Y mutation in *pfmdr1* that had been shown to modulate CQR was found in 67.1% of the samples positive for the CVIET haplotype.

**Conclusions:**

This is the first report that details malaria epidemiology and also the molecular status of CQ-resistance in *P. falciparum* population of the region. The efforts of the State Vector Borne Disease Control Programme have proved to be quite effective in controlling the malaria burden in the state. Despite the discontinuation of CQ for a decade, local *P. falciparum* is observed with decreased CQ-sensitive haplotype. It is believed that the present findings will form a basis for further studies on genetic diversity in *P. falciparum,* which could confer better understanding of the complexity of the disease in Southeast Asia.

## Background

Malaria transmission in India occurs predominantly either as *Plasmodium falciparum* or *Plasmodium vivax* infections. India and 15 sub-Saharan African countries are estimated to carry 80% of malaria burden across the world in 2018 [1]. In India, 0.84 million malaria cases were reported in 2017, of which North Eastern (NE), Eastern and Central Indian regions contributed 80% of the total cases [2]. Mizoram, along with Assam, Arunachal Pradesh, Tripura, Manipur, Mizoram, Sikkim and Meghalaya form the eight NE States of India. NE India accounts for 4% of the population but contributed 6.6% of malaria cases and 25% of malaria mortality in India in 2018 [3].

Mizoram is a landlocked state in NE India with an estimated population of 1 million in the 2018 Census. Mizoram extends between latitude 21°58′N to 24°35′N and longitude 92°15′E to 93°29′E, and altitude ranges from 500 to 2157 m. Mizoram is under the direct influence of southwest monsoon characterized by wet summers (18–33 °C) and dry winters (11–24 °C) [4]. The state lies within the Indo-Burma region, one of the four biodiversity hotspots of India. The eastern and western regions of Mizoram share international borders with Myanmar and Bangladesh, respectively and shares domestic borders with Manipur, Assam and Tripura. The porous border with Myanmar could be one of the major entry routes of drug-resistant parasites to mainland India.

In 1973, the first cases of *P. falciparum* chloroquine resistance (CQR) were reported from Karbi Anglong district in Assam [5]. In the early 1980s, CQR was reported in Mizoram [6]. Most likely, CQR has spread from Southeast Asia (SEA) to the Indian mainland through NE India [7]. Treatment failures to sulfadoxine/pyrimethamine (SP) were first reported in NE India in 2005 [8] and molecular evidence to its resistance in *P. falciparum* was reported in 2013 [9, 10]. This ushered in a change in the national drug policy of India; artesunate plus sulfadoxine-pyrimethamine (SP) has been replaced by a co-formulated tablet of artemether-lumefantrine (AL) in NE India since 2013 [11].

Within the 72 to 76 amino acid loci of *P. falciparum* chloroquine resistant transporter gene (*pfcrt*), the chloroquine-sensitive (CQS) strains have been marked with CVMNK allele [12], while polymorphism within this locus conferring CQR [13] is characterized by CVIET and CVIDT in parts of SEA and Indochina, respectively [14], SVMNT in Africa [15], and CVMNT in South America [16, 17]. In addition, 32-point mutations in *pfcrt* have been identified to date and of these 11 are associated with CQR. Worldwide, K76T is considered to be the major molecular marker and a key determinant of CQR [18]. CQR, mediated by *pfcrt* mutations, has been shown to be modulated by mutations in *P. falciparum* multidrug resistance gene-1 (*pfmdr1*). Among the five (N86Y, Y184F, S1034C, N1042D, D1246Y) *mdr1* mutations, N86Y and Y184F have been linked with CQR [19–21].

Because the molecular status of CQR in *P. falciparum* in Mizoram remained poorly documented, the present study aimed at screening for polymorphisms within the genes linked to CQR (*pfcrt* and *pfmdr1*) across Mizoram. Additionally, the study aimed to present malaria epidemiology from 2010 to 2018. Due to the discontinuation of CQ as the first-line *P. falciparum* treatment in 2008, it was expected that the prevalence of CQS strain would be more abundant than CQR haplotype.

## Methods

### Epidemiology data

**Fig. 1:**
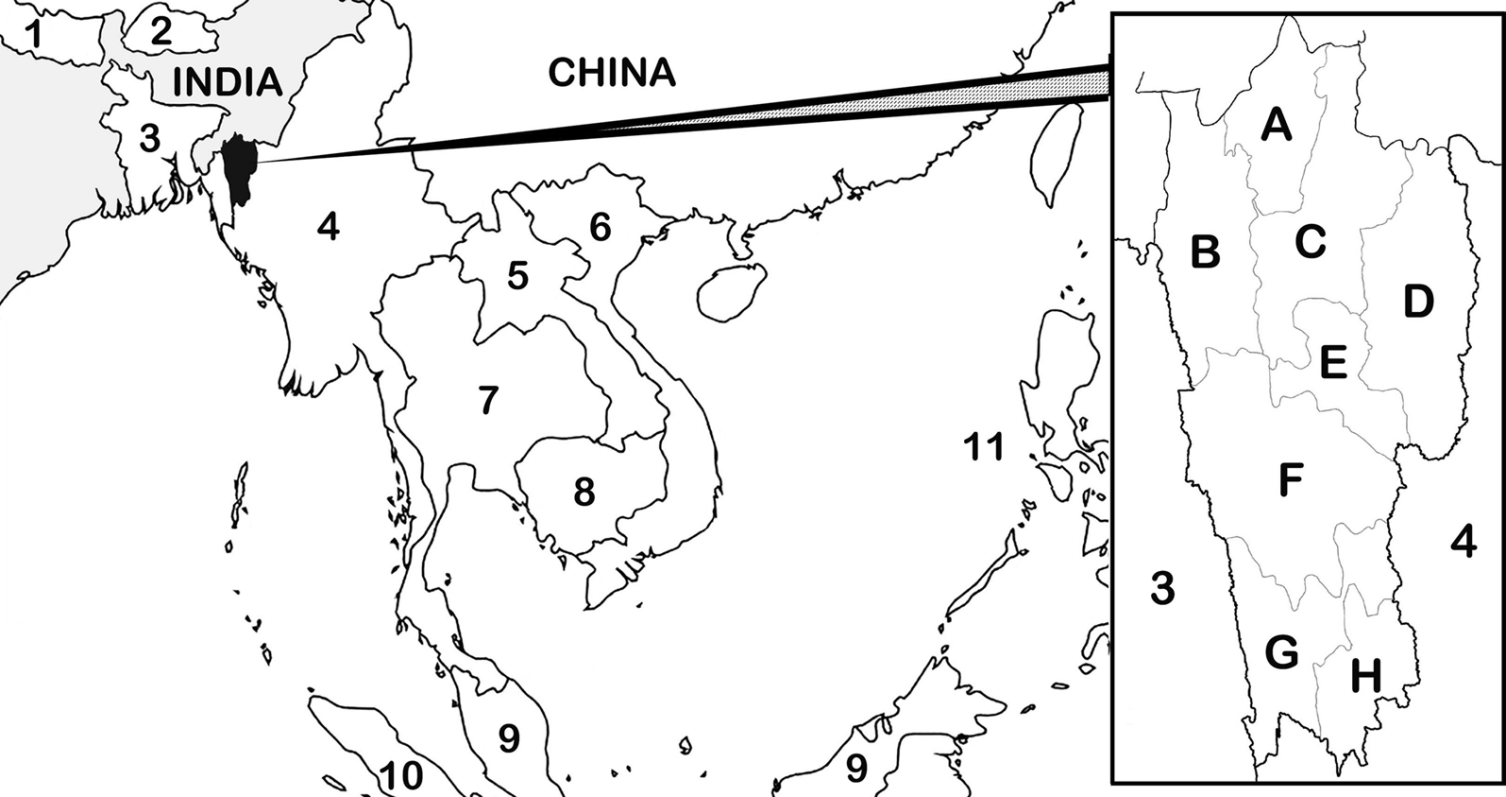
Map of Mizoram, India illustrating its proximity to Southeast Asian countries. A Kolasib district, B Mamit district, C Aizawl district, D Champhai district, E Serchhip district, F Lunglei district, G Lawngtlai district, H Saiha district. 1 Nepal, 2 Bhutan, 3 Bangladesh, 4 Republic of the Union of Myanmar, 5 Laos, 6 Vietnam, 7 Thailand, 8 Cambodia, 9 Malaysia, 10 Indonesia, 11 Philippines

Mizoram is divided into 8 districts: Aizawl, Lunglei, Kolasib, Mamit, Lawngtlai, Champhai, Saiha, and Serchhip (Fig. 1). Available monthly and annual records of malaria (*P. falciparum* and *P. vivax*), including the number of deaths due to malaria (*P. falciparum* and *P. vivax* combined) and anti-malarial drug treatment for *P. falciparum* were collected from the office of the State Vector Borne Disease Control Programme (SVBDCP) for the 8 districts of Mizoram from 2010 to 2018 (Fig. 1). Malaria case distribution was further categorized into gender (proportion of males and female), age (0–4 years, 5–14 years and 15 + years; as recorded and available in SVBDCP records), passive case detection (PCD) i.e., febrile patients suspecting malaria seeking medical confirmation), and pregnancy infections.

### Sample collection

From 2015 to 2017, confirmed *P. falciparum*-positive blood samples were collected from government district hospitals, community health centres, primary health centres, sub-centres, and diagnostic centres of Aizawl, Lunglei, Saiha, Kolasib, Mamit, Lawngtlai, and Serchhip districts (Champhai district was excluded for sample collection due to its consistently low annual parasite incidence (API) (Fig. 1). Samples were collected after obtaining consent from patients and guardians. For patients under 10 years of age, consent was obtained from their guardians (i.e., either one or both parents present at the time of blood sampling). Details regarding new or relapse infection, age and gender of the patient, date of blood examination and collection of samples; onset and duration of anti-malarial drug treatment administered were recorded for each blood sample collected. Blood samples were spotted on filter paper or were collected in EDTA vials, and preserved at − 20 °C for further analysis.

### DNA extraction and species-specific identification of malaria parasite

QIAamp Mini Kit (QIAGEN) was used to extract genomic DNA from positive *P. falciparum* samples according to manufacturer protocol. The extracted DNA was stored at − 20 °C prior to use. Confirmatory PCR to detect *P. falciparum* and *P. vivax* was performed by targeting the 18SrRNA region as previously described [22].

### PCR amplification and sequencing

The details used for *pfcrt* and *pfmdr* are given in Additional file 1: Table S1. For *pfcrt*, three nested PCRs were performed using gene-specific primers to detect polymorphism at exons 1–2, 2–8 and 9–12. The primary PCR was carried out in 25 µl volume using EmeraldAmp GT PCR mix (Takara Bio Inc., Japan), 0.1 µM primers and 1–3 µl of DNA template. The PCR conditions were 94 °C for 5 min, 35 cycles of 92 °C for 30 s, 50 °C for 40 s, 62 °C for 1 min and a final extension at 62 °C for 5 min. For the nested PCR, the primary PCR product was diluted 10 times and 1 µl was used as a template for 50 µL reaction volume. The nested PCR conditions were 94 °C for 4 min, 35 cycles of 92 °C for 1 min, 55 °C for 40 s, 72 °C for 1 min and a final extension step of 72 °C for 5 min. The PCR products (40 µL approx.) were purified from agarose gel using QIAquick Gel Extraction Kit (QIAGEN) according to manufacturer protocol and then immediately sent for sequencing. *Pfmdr1* PCR was carried out in 25 µl volume using EmeraldAmp GT PCR mix (Takara Bio Inc., Japan), 0.1 µM primers and 1–3 µl of DNA template. For amplifying codons 86 and 184, the PCR conditions were 94 °C for 5 min, and 35 cycles of 94 °C for 30 s, 52 °C for 30 s, 72 °C for 1 min and a final extension at 72 °C for 5 min. For codons 1034, 1042 and 1246, the annealing temperature used was 56 °C for 30 s. The PCR products were preserved at − 20 °C prior to sequencing.

The PCR amplicons were sequenced at DBT- State Biotech Hub, Dept of Biotechnology, Mizoram University, Aizawl, Mizoram and Agrogenome, Cochin, Kerala, India.

## Data analysis

### Epidemiological data

The following parameters were calculated:- total blood examined (TBE = total blood slides examined + total rapid diagnostic test (RTD) conducted; if an individual was tested for both blood slides and RTD, blood slide was considered), total blood positive for malaria (TBM = blood slides + RDT; both positive for malaria), surveillance coverage (SC = TBE/overall fever cases × 100), annual blood examination rate (ABER = TBE/population ×100), slide positive rate (SPR = TBM/TBE ×100), per cent *P. falciparum* (% Pf = blood positive for *P. falciparum*/TBM ×100), per cent *P. vivax* (% Pv = blood positive for *P. vivax*/TBM ×100).

### Nucleotide and amino acid data

The *pfcrt* and *pfmdr1* nucleotide and amino acid sequences were aligned using MAFFT [23] and MEGA 7 [24]. The *pfcrt* sequences were aligned with 3D7 CQS strain (GenBank Accession No. AL844506.3:403222-406317) to detect polymorphisms at 11 codon positions: 72 (C/S), 74 (M/I), 75 (N/E), 76 (K/T), 152 (T/A), 163 (S/R), 220 (A/S), 271 (Q/E), 326 (N/S), 356 (I/T) and 371 (R/I). *Pfmdr1* sequences were aligned with the reference 3D7 sequence (GenBank Acc. No. NC 004326:957885-962144), and were analysed for point mutations at positions 86 (N/Y), 184 (Y/F), 1034 (S/C), 1042 (N/D) and 1246 (D/Y).

### Statistical analysis

Epidemiological and molecular data were entered, analysed in Excel (Microsoft) and graphical presentations were also performed in Excel. Association between *pfcrt* and *pfmdr1* mutation was analysed using Chi square test in Statistical Package for Social Science (IBM SPSS Statistics for Windows, Version 24.0. IBM Corp., Armonk, NY, USA).

## Results

### Malaria epidemiology analysis

**Fig. 2:**
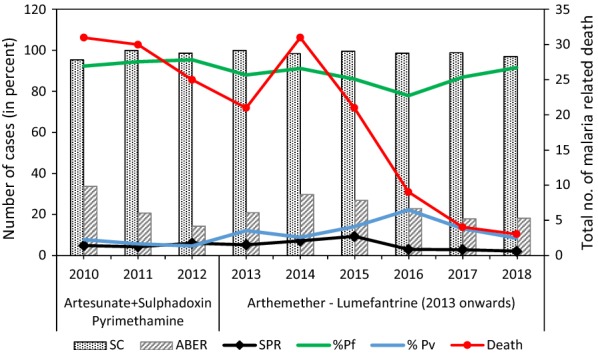
Overall summary of malaria epidemiology for 9 years (2010–2018) highlighting malaria-related deaths and the treatment used for *Plasmodium falciparum* cases. *SC* surveillance coverage, *ABER* annual blood examination rate, *SPR* slide positive rate,  *%Pf* per cent of blood positive for *P. falciparum*,  *%Pv* per cent of blood positive for *P. vivax*. (The calculations for these parameters are explained in the “Methods” section)

Malaria epidemiology recorded during 2010–2018 showed that malaria was persistent throughout the year, and across all districts in the state. The surveillance coverage accounted for ≥ 95% in all sites of study (Fig. 2). Majority of malarial infection and malaria-related death was due to *P. falciparum* infection and accounted for 89.3% of total malaria cases (Fig. 2 and Additional file 3: Table S3). The observed highest malaria incidence and malaria-related deaths was in 2014. Reason for the high mortality rate in 2014 was because the majority of infection had evolved to hyper-reactive malarial splenomegaly. The factors were: (i) lack of timely detection of the parasite due to lack of transportation (mainly roadblock due to landslides); and, (ii) less availability of *P. falciparum* treatment (ACT-AL) in almost all rural medical facilities; patients were devoid of immediate medical assistance. However, a decline in the number of malaria-related deaths was observed in subsequent years (Fig. 2). The total number of death due to malaria was 172; Mamit and Lawngtlai recorded deaths annually throughout the study period (Additional file 3: Table S3).

**Fig. 3:**
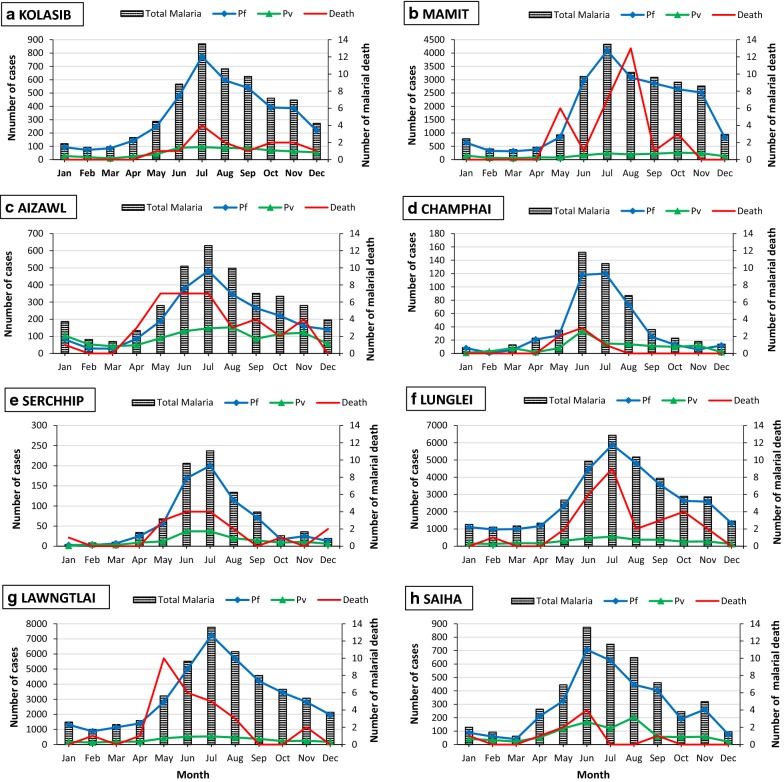
Monthly distribution of malaria cases and malaria-related deaths (2010–2018) at each study site (**a**–**h**)

Overall, most malaria incidence was contributed by Lawngtlai (average API_2010–2018_ = 34.8; total number of malaria cases = 41,516; number of *P. falciparum *= 37,892; number of *P. vivax* = 3664); followed by Mamit (average API_2010–2018_ = 34.4; total malaria cases = 23,391; *P. falciparum *= 21,497; *P. vivax* = 1894). The total sum of malaria cases (total malaria, total *P. falciparum*, *P. vivax* infections and death) throughout 9 years of study was calculated. This was an attempt to observe the trend of infection and to find any discrepancy in its abundance at monthly resolution (Fig. 3). A consistent and cyclic pattern of gradual increase in malaria cases during the rainy summer/monsoon season (March to September) was observed. The cases peaked in June to July and then decreased during the dry winter season (October to January) (Fig. 3). A similar pattern was also observed for the number of malaria-related deaths in all study areas, excluding Mamit and Lawngtlai. Maximum deaths were reported from Mamit (n = 13) during August.

Distribution of age and gender-specific malarial infection, passive case detection and pregnancy infections recorded for 2014 to 2018 were analysed (Table 1). Overall, 15 + years of age had the highest malaria incidence (39,407/69,178), whereas ≤ 4 years of age had the lowest (9047/69,178). Males of 15 + years contributed the highest with 35.9% of total cases (Table 1, Additional file 3: Table S3). The total number of pregnancy infections recorded from 2014 to 2018 was 364 (0.53% total malaria cases), and deaths during pregnancy were not reported. A total of 61,501 individuals were recorded for passive case detection (PCD).

**Table 1:** Distribution of malaria cases during 2014 to 2018

Study site	Year	≤4 yrs			5–14 years			15 + years			Preg.	PCD	Study site	Year	≤4 years.			5–14 years			15 + years			Preg.	PCD
		M	F	Total	M	F	Total	M	F	Total					M	F	Total	M	F	Total	M	F	Total		
		%	%		%	%		%	%		Total	Total			%	%		%	%		%	%		Total	Total
A.	2014	66.7	33.3	27	63.6	36.4	99	75.7	24.3	738	4	835	E.	2014	75.0	25.0	4	76.2	23.8	21	68.0	32.0	153	0	177
	2015	56.9	43.1	72	59.7	40.3	134	74.2	25.8	730	2	863		2015	80.0	20.0	5	58.3	41.7	12	81.3	18.8	144	0	154
	2016	41.2	58.8	17	62.8	37.2	43	72.4	27.6	170	1	211		2016	0.0	100	4	83.3	16.7	6	82.4	17.6	34	1	44
	2017	75.0	25.0	4	70.6	29.4	17	83.7	16.3	98	1	103		2017	0.0	0.0	0	0.0	0.0	0	77.8	22.2	9	0	8
	2018	66.7	33.3	3	57.1	42.9	7	88.5	11.5	26	0	35		2018	0.0	0.0	0	50.0	50.0	2	100	0.0	6	0	4
B.	2014	49.9	50.1	676	53.7	46.3	1557	64.5	35.5	2833	30	4339	F.	2014	49.1	50.9	1049	53.7	46.3	1724	59.5	40.5	3247	16	5651
	2015	51.4	48.6	1277	53.6	46.4	2711	65.2	34.8	4678	58	6525		2015	49.2	50.8	1228	47.3	52.7	2442	61.9	38.1	4050	119	7141
	2016	50.2	49.8	241	55.4	44.6	531	65.8	34.2	927	8	1370		2016	43.2	56.8	301	49.0	51.0	747	59.9	40.1	1274	9	2198
	2017	48.8	51.2	82	49.4	50.6	239	64.7	35.3	464	4	679		2017	53.2	46.8	190	54.2	45.8	441	61.7	38.3	848	5	1397
	2018	46.6	53.4	103	57.1	42.9	273	57.3	42.7	396	3	589		2018	51.3	48.7	113	46.5	53.5	353	60.5	39.5	626	12	1036
C.	2014	31.0	69.0	42	38.8	61.2	188	76.4	23.6	993	7	1081	G.	2014	49.3	50.7	1172	50.5	49.5	2950	58.3	41.7	4756	19	8564
	2015	48.6	51.4	35	46.2	53.8	119	80.1	19.9	1182	7	1054		2015	50.5	49.5	1097	50.9	49.1	2795	58.2	41.8	4594	20	7594
	2016	44.4	55.6	18	60.5	39.5	38	72.6	27.4	365	1	364		2016	50.7	49.3	367	49.8	50.2	851	59.5	40.5	1288	5	2297
	2017	0.0	100.0	1	66.7	33.3	18	77.3	22.7	132	1	121		2017	41.6	58.4	423	48.0	52.0	1019	57.2	42.8	1532	1	2776
	2018	50.0	50.0	2	54.5	45.5	11	75.0	25.0	44	0	43		2018	56.9	43.1	295	49.1	50.9	753	57.1	42.9	1174	21	2070
D.	2014	100	0.0	1	83.3	16.7	12	76.8	23.2	69	0	62	H.	2014	50.0	50.0	76	51.3	48.7	189	66.2	33.8	557	0	694
	2015	66.7	33.3	3	37.5	62.5	8	52.5	47.5	61	2	51		2015	47.5	52.5	59	58.7	41.3	254	67.2	32.8	774	5	821
	2016	0.0	0.0	0	14.3	85.7	7	77.8	22.2	18	0	20		2016	51.4	48.6	35	52.2	47.8	67	65.2	34.8	221	2	292
	2017	0.0	0.0	0	0.0	0.0	0	66.7	33.3	6	0	6		2017	36.8	63.2	19	56.7	43.3	60	63.7	36.3	113	0	153
	2018	0.0	0.0	0	100	0.0	1	80.0	20.0	5	0	5		2018	83.3	16.7	6	84.0	16.0	25	75.0	25.0	72	0	74
Total number of malaria cases (all districts)										≤ 4 years				5–14 years				≥ 15 years				Preg.		PCD	
										M		F		M		F		M		F					
										4488		4559		10637		10087		24836		14,571		3641		61,501	
Grand total (excluding Preg. and PCD)										69,178															
% Total (Sex + Age)										6.49		6.59		15.38		14.58		35.90		21.06					

A Kolasib, B Mamit, C Aizawl, D Champhai, E Serchhip, F Lunglei, G Lawngtlai, H Saiha

*M* male, *F* female, *Preg* infection during pregnancy, *PCD* passive case detection

### Molecular analysis of *pfcrt* and *pfmdr1* for CQR mutation

**Fig. 4:**
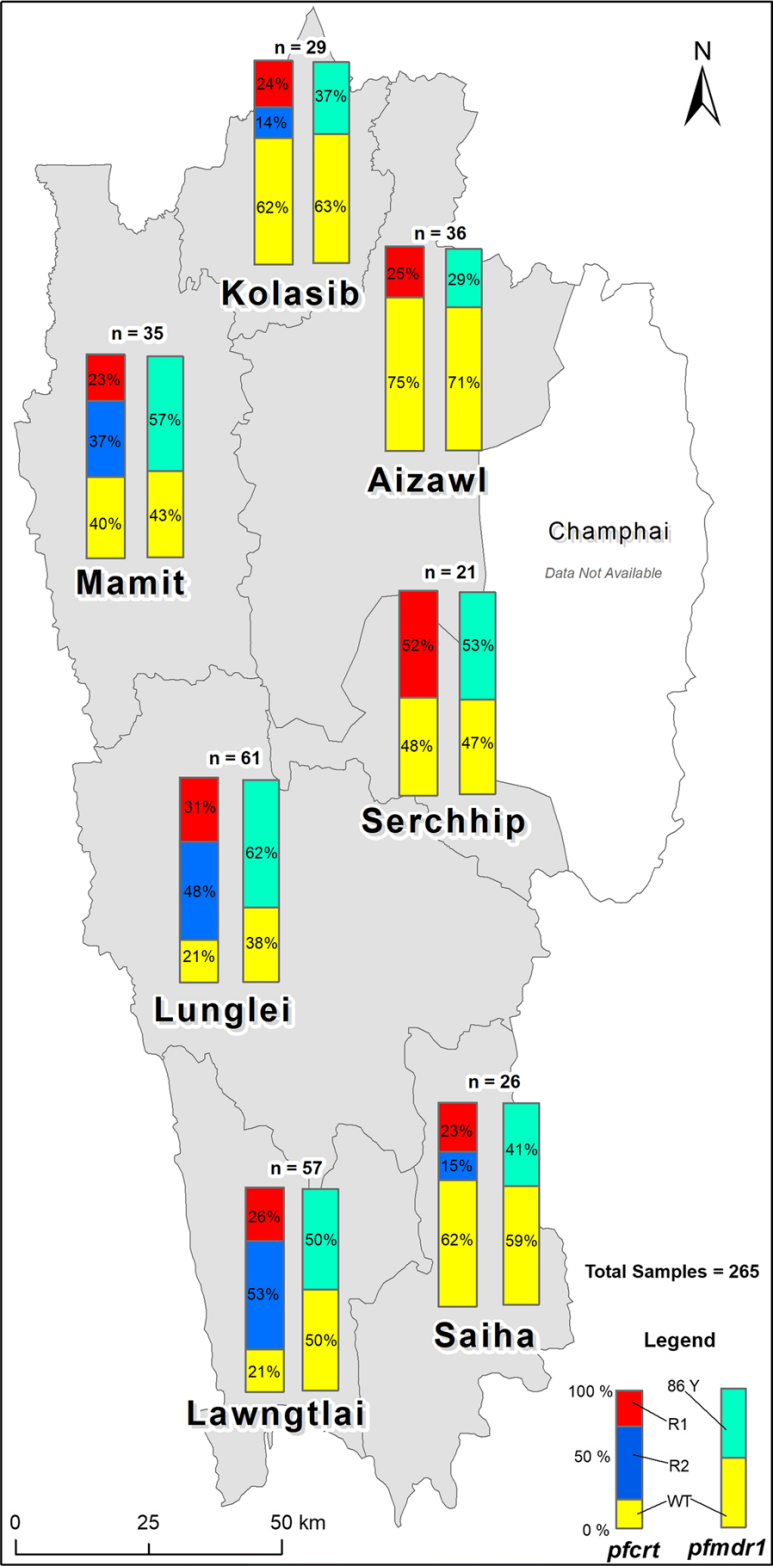
Distribution of chloroquine resistance transporter (*pfcrt*) and multidrug-resistant (*pfmdr1*) genotypes across Mizoram. *WT* wild type, *R* CQR genotype, *R1* CQR type-1, *R2* CQR type-2; 86Y: N → Y substitution at 86 amino acid of *pfmdr1*; All calculations are in percentages

Previously characterized *pfcrt* mutations in 11 amino acid loci 73–76, 152 and 163, 220, 271, 326, 356 and 371 conferring CQR were analysed in 265 *P. falciparum*-infected clinical blood samples from 2015 to 2017. 3D7 strain was used as a reference. Some 41.5% (110/265) of the samples displayed sequence similarity with the reference sequence, and were denoted as ‘WT’ (wild type). The remaining 58.5% (155/265) of the samples displayed substitutions at 7 amino acid positions (CQR mutations) and were distributed throughout the study sites (Table 2). These samples are denoted as ‘R’. All the R samples were positive for the Southeast Asian CVIET *pfcrt*-resistant haplotype. Within the R populations, because of additional amino acid polymorphism at positions 220 (A → S), 271 (Q → E) and 371 (R → I), a further subdivision was made into ‘R1’ (IET-AQ-S-R) and ‘R2’ (IET-SE-S-I) (Table 2). R1 and R2 constituted 48.4 and 51.6% of the total CQR mutated populations, respectively. The distribution of WT and R (R1 and R2) among the 7 districts of Mizoram is given in Fig. 4. WT, R1 and R2 were not uniformly distributed within Mizoram; WT was observed highest in Aizawl with 75% of the total sample analysed (26/35), followed by Kolasib (62%,18/29) and Saiha (61%, 16/26). Lawngtlai, Lunglei and Mamit showed a higher prevalence of R forms; 78.9% (45/57), 78.7% (48/59) and 60% (21/35), respectively (Fig. 4). Within R populations, Aizawl and Serchhip were positive only for R1; mixed populations of R1 and R2 were observed in Lunglei, Saiha, Kolasib, and Lawngtlai. R2 was highest in Lawngtlai (52%) followed by Lunglei (47.5%) (Fig. 4). All the *pfcrt* nucleotide sequences were deposited to GenBank (see Additional file 2: Table S2).

**Table 2:** Pattern of *pfcrt* amino acid sequence diversity of at seven amino acid positions

Exon number			2	2	2	4	6	9	11
Amino acid loci and substitution			74	75	76	220	271	326	371
Study sites		*N (nR)*	M/I (%)	N/E (%)	K/T (%)	A/S (%)	Q/E (%)	N/S (%)	R/I (%)
A. Kolasib		29	M (62.1)	N (62.1)	K (62.1)	A (86.2)	Q (86.2)	N (62.1)	R (86.2)
		(11)	I (37.9)	E (37.9)	T (37.9)	S (13.8)	E (13.8)	S (37.9)	I (13.8)
B Mamit		35	M (40.0)	N (40.0)	K (40.0)	A (62.9)	Q (62.9)	N (40.0)	R (62.9)
		(21)	I (60.0)	E (60.0)	T (60.0)	S (37.1)	E (37.1)	S (60.0)	I (37.1)
C. Aizawl		36	M (75.0)	N (75.0)	K (75.0)	A (100)	Q (100)	N (75.0)	R (100)
		(9)	I (25.0)	E (25.0)	T (25.0)			S (25.0)	
E. Serchhip		21	M (47.6)	N (47.6)	K (47.6)	A (100)	Q (100)	N (47.6)	R (100)
		(11)	I (52.4)	E (52.4)	T (52.4)			S (52.4)	
F. Lunglei		61	M (21.3)	N (21.3)	K (21.3)	A (52.5)	Q (52.5)	S (78.7)	R (52.5)
		(48)	I (78.7)	E (78.7)	T (78.7)	S (47.5)	E (47.5)	N (21.3)	I (47.5)
G. Lawngtlai		57	M (21.1)	N (21.1)	K (21.1)	A (47.4)	Q (47.4)	N (21.1)	R (47.4)
		(45)	I (78.9)	E (78.9)	T (78.9)	S (52.6)	E (52.6)	S (78.9)	I (52.6)
H. Saiha		26	M (61.5)	N (61.5)	K (61.5)	A (84.6)	Q (84.6)	N (61.5)	R (84.6)
		(10)	I (38.5)	E (38.5)	T (38.5)	S (15.4)	E (15.4)	S (38.5)	I (15.4)
3D7 Reference sequence			M	N	K	A	Q	N	R
Genotypes									
1. WT			M	N	K	A	Q	N	R
2. R1			I	E	T	A	Q	S	R
3. R2			I	E	T	S^a^	E^a^	S	I^a^

*n* total number of samples, *nR* number of samples with R forms, *WT* wild type, *R1* CQR type-1, *R2* CQR type-2

^a^Denotes additional substitutions; All calculations are in percentage

A total of 356 samples were sequenced and all nucleotide sequences were deposited to GenBank (see Additional file 2: Table S2). Table 3 shows the analysis of *pfmdr1* mutations obtained from these study areas. Analysis for mutation at amino acid 86 position showed that 174 samples (48.9% of the total sample) displayed the N86Y mutation (Table 3). However, amino acid sequences displayed no polymorphisms at 184 (Y/F), 1034 (S/C), 1042 (N/D) and 1246 (D/Y). The highest (63.2%) and lowest (28.6%) number of N86Y mutations were observed in samples from Lunglei and Aizawl, respectively. Lawngtlai showed uniform distribution of N86 and 86Y (Table 3).

**Table 3:** Analysis of *pfmdr1*

Study sites	*N*	N86Y (%)	Y184F (%)	S1034C (%)	N1042D (%)	D1246Y (%)
Kolasib	32	N (63.6); Y (36.4)	Y184 (100)	S1034 (100)	N1042 (100)	D1246 (100)
Mamit	44	N (43.5); Y (56.5)				
Aizawl	42	N (71.4); Y (28.6)				
Serchhip	86	N (46.7), Y (53.3)				
Lunglei	74	N (36.8); Y (63.2)				
Lawngtlai	46	N (50.0), Y (50.0)				
Saiha	32	N (59.4); Y (40.6)				

*N* number of samples analysed. (All calculations are in percentage)

The result of Chi square analysis in 265 samples tested for both *pfcrt* and *pfmdr1* polymorphisms showed a significant association existed between the mutations (χ^2^ = 121.48, df = 1, p = 0.001, ϕ_c_ = 0.68). The key 86Y mutation in *pfmdr1* that had been shown to modulate CQR was found in 67.1% of R-population of *pfcrt* gene (n = 155) analysed, meanwhile its mutation was found to be nil in WT-populations of *pfcrt* (n = 110) (Fig. 4).

## Discussion

Historically the spread of CQR has been linked from SEA to Africa via the Indian subcontinent [25, 26], and NE states could have contributed to CQR spread in mainland India through host movements [7]. The eastern region of Mizoram shares its international border with Myanmar. By land, one can enter Tedim in the Chin state of Myanmar through the Zokhawthar-Rih border of Champhai district. Both regions share a historical similarity in ethnic, religion, culture, and language [27]. Trade relations between the locals in the border areas have led to continuous movement of people in both directions. Despite the strategic location of Mizoram, and its importance as a conduit for the spread of drug-resistant *P. falciparum*, malaria epidemiology and drug resistance status have been poorly documented.

This is the first study to give a detailed account of malaria epidemiology in Mizoram in this decade. *Plasmodium falciparum* contributes to the majority of the cases (> 89%) in Mizoram, similar to that observed in its national borders [1]. A study carried out in the early 1980 s incriminated *Anopheles minimus* and *Anopheles dirus* as the major vectors in Mizoram [28, 29]. Additionally, recent studies on vector distribution identified *Anopheles campestris*, *Anopheles jeyporiensis*, *Anopheles maculatus*, and *Anopheles nivipes* as the most abundant species in malaria endemic parts of Mizoram [30, 31]. It has also been documented that the rise in *Anopheles* populations is subsequently followed by a peak in malaria cases [31]. In all districts of Mizoram, malaria cases peak during the major monsoon season: May to July (Fig. 3). Malaria transmission is perennial and cases are reported throughout the year; the burden is highest in the districts of Lunglei, Lawngtlai and Mamit. Champhai, which links with Myanmar through an international highway, consistently reported the least API score (Additional file 3: Table S3). The low parasite prevalence could be attributed to its high elevation (1678 m above sea level), in parallel with low abundance of anopheline species in the region. Even though Mizoram is a high malaria-endemic region, a gradual decrease in the number of *P. falciparum* cases and mortality due to malaria has been observed from 2010 onwards. One important factor could be attributed to the current *P. falciparum* treatment (ACT-AL), and to the strategies implemented by the SVDCP that includes: visual and verbal awareness campaigns about the disease, and about potential breeding habitats, outdoor residual spraying of DDT twice a year, and quinquennial distribution of > 500,000 deltamethrin-treated bed nets. This is evidenced by the substantial number of PCD seeking medical care (Table 1). Furthermore, efficient and easy access to healthcare facilities could contribute to effective management and timely treatment of pregnancy infections as not a single pregnancy death due to malaria has been reported during the study.

This is the first report conducted to determine the molecular status of CQR in Mizoram; the study targeted the established molecular markers of *pfcrt* and *pfmdr1*. Artesunate + SP is currently the treatment for uncomplicated *P. falciparum* in all parts of India, except NE India. It has been a decade since the use of CQ for *P. falciparum* cases; the change from CQ to ACT-AL as first-line treatment was expected to result in wide spread distribution of CQS allele within the *P. falciparum* population, as documented in many African countries [32–36]. Unexpectedly, only 41.5% (110/265) of the samples were identified as CQS type in the study sites (Fig. 4). Perhaps this is because CQ (the current the treatment for *P. vivax* infection) could be employed for treating unrecognized mixed infections, thereby making *P. falciparum* vulnerable to the drug [37]. In addition, the present study on *pfcrt* polymorphisms indicated that CQS (41.5%) and CQR (58.5%) co-existed, albeit when CQ treatment ceased, in a manner similar to other parts of Asia [38, 39] (Fig. 4). In India, the major *pfcrt* haplotype identified is the SVMNT; yet, varying frequencies of CVIET, CVIDT and CVMNT have also been reported [40–44]. Interestingly, in Mizoram was observed with 58.5% (155/265) of the samples were positive for CVIET allele, and is the only haplotype available in the region; CVIET is the predominant CQR-allele found mainly in SEA. The *P. falciparum* population, having the CVIET allele was further divided into two groups: R1 (CVIET-AQ-S-R) and R2 (CVIET-SE-S-I); R2 contains amino acid substitutions similar to Old World-resistant phenotype [45, 46] (Table 2). Subsequent in vitro studies on R1 and R2 of local *P. falciparum* populations may give rise to further prospective study on CQ-sensitivity in the future.

Globally, five-point mutations (N86Y, Y184F, S1034C, N1042D and D1046) have been described for *pfmdr1* and, importantly, 86Y polymorphism is linked with decreased sensitivity to CQ [47] and amodiaquine (chemically similar to CQ) [48, 49]. It has been recorded that the N terminal, N86Y and Y184F mutations are more prevalent in African and Asian parasites, while the C terminal mutations: S1034C, N1042D and D1046Y are more common in South American parasites [15]. This study identified a single 86Y substitution at the N terminal region with 48.9% (174/356) while the remaining 51.1% (182/356) displayed uniformity throughout the amino acid sequence. Substitutions at 184 and the later three codon positions discussed above were not observed. Analysis indicates that 67.1% of samples with CVIET haplotype also carried N86Y mutation. A similar pattern had been observed in parts of India [37], Asia [38, 50–52] and Africa [35, 53, 54].

## Conclusion

Malaria is perennial throughout Mizoram and *P. falciparum* transmission accounts for more malaria cases than *P. vivax*. However, the efforts of SVDCP have proved to be quite effective in controlling the malaria burden in the state, reducing malaria transmission and malaria deaths over the years. The only available CQR allele found across the state is the South Asian CVIET haplotype. Despite the discontinuance of CQ for a decade, polymorphisms at *pfcrt* (76T) and *pfmdr1* (86Y) continued to exist at an unexpected rate. In addition, a single polymorphism at site 86 of *pfmdr1* proved to be a unique characteristic feature of local resistant *P. falciparum* population. It is speculated that less abundance of mutated *pfmdr1* 86 (N → Y) substitution and absence of polymorphism at codon 184, 1034, 1042 and 1046 loci may still contribute to high efficacy in artemisinin-lumefantrine treatment currently employed within the region. Further studies on genetic diversity of local *P. falciparum* in greater detail would confer better understanding about the complexity of the disease.

## Supplementary information

**Additional file 1: Table S1.** Details of primers used in the study.

**Additional file 2: Table S2.** Voucher ID of samples and their respective GenBank Accession Numbers.

**Additional file 3: Table S3.** . Detailed analysis on epidemiological data.

## Data Availability

The datasets during and/or analysed during the current study available from the corresponding author on reasonable request.

## Abbreviations

*Pfcrt*: *P. falciparum chloroquine resistant transporter*
*pfmdr1*: *P. falciparum multidrug resistance 1*
SEA: Southeast Asia
NE: North East India
SVDCP: State Vector Disease Control Programme
NVBDCP: National Vector Borne Disease Control Programme
CQ: Chloroquine
CQR: Chloroquine resistance
SP: Sulfadoxine-pyrimethamine
ACT: Artemisinin-based combination therapy
AL: Artemether-lumefantrine
